# Cancer patients with large defects. Reconstructional options: A case study

**DOI:** 10.1016/S1808-8694(15)31290-8

**Published:** 2015-10-20

**Authors:** Theodoros Papadas, Panagiotis Goumas, Maria Miranda Alexopoulou, Ioannis Papakyriakos, Dimitrios Papavasiliou, Dimitrios Antonopoulos

**Affiliations:** 1 Department of Otorhinolaryngology, University Hospital of Patras; 2 Department of Oral Maxillofaciall Surgery, University Hospital of Patras; 3 Plastic Surgery Clinic, Saint Andrews Hospital of Patras

**Keywords:** squamous cell carcinoma, esthetical and functional rehabilitation, metal fixed prosthesis, myocutaneous *trapezious fla p*

## Abstract

We report a case of a seventy-five years old male patient with a squamous cell carcinoma (SCC) originated from the right external ear four years ago. He was undergone surgical removal of the lesion with a combination of modified neck dissection and reconstruction with the use of pectoralis major flap. Furthermore, he had radiotherapy with 6000 rads of the right temporal region. Two months ago the patient showed an extended recurrence concerning the temporal muscle and bone, the lithoid bone, the maseter and the pterygoids muscles, the right part of the mandible, the parotid gland with the facial nerve, and the superior bulb of the internal jugular vein. He had a surgical removal of the lesion in extended healthy margins and functional and esthetic reconstruction of the defect with a combination of metal fixed prosthesis of the condyle and the right mandible and the use of myocutaneous *trapezious fla p*. This is a case report of the reconstruction options we have nowadays to provide quality of life in cancer patients.

## INTRODUCTION

Squamous cell carcinoma (SCC) is a malignant tumor of the face affecting soft tissues as well as bone structures with high frequency of metastases. Cancer patients with SCC of the skull and the facial skeleton have undergone extended surgeries with combination of preoperative or postoperative chemo and radiotherapy with very good results and high rates of survival.

Sometimes lesions are very extended due to topical recurrence or because of negligence, leaving large defects after the surgical removal, and skin invasion is often present.

The main problem is the functional and esthetical rehabilitation of these patients, providing them with a quality of life. Nowadays the options we have are numerous, using combinations of flaps with fixed prosthesis and bone grafts.

There are several reports in the literature on the therapeutic results after surgical removal of malignant tumors of the region^1^, ^2^, ^3^ and also reports which review reconstructive results using flaps such as pectoralis major, trapezious, latissimus dorsi or free flaps^4^, ^5^, ^6^ but very few reports concerning the reconstruction defects of the parotid area^5^, ^6^, ^7^, ^8^.

The aim of this paper is to describe our experience of the use of myocutaneous flaps and combination of them with reconstructive plates for covering large defects.

## CASE REPORT

A 75-year-old man showed in our clinic four years ago with a lesion of the right auricle. After X-rays, C/T examinations and biopsy of the lesion he underwent a surgical removal of the lesion concerning the whole right external ear, with combination of modified neck dissection. The ear defect was covered with the use of the right pectoralis major flap, and the histological results showed a SCC carcinoma of medium differiation. A year after, during the regular follow-up examination, a recurrence of the 1cm × 0,5cm lesion was revealed.

Under general anesthesia, a modified right mastoidectomy was performed, and postoperatively the patient had a course of 6000 rads of radiotherapy in the right temporal region.

There were no complications postoperatively and he had a two-year uneventful follow-up. Eight months ago, and while the patient had not showed up for a year, he came back complaining of severe trismus, otorrhea, pus of the external auditory canal and the right temporal bone, bone necrosis due to radiotherapy and right facial palsy.

**Figure 1:**
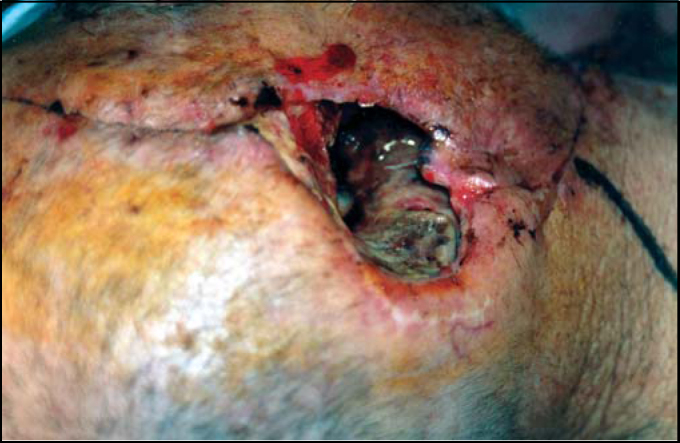
Clinical view of the defect at the right external auditory canal

The clinical examination revealed a large defect (Figure 1), and after C/T examination, he underwent radical mastoidectomy, lateral temporal bone resection, total parotidectomy with resection of the right facial nerve and right partial mandibulectomy.

The skin in the area was invased concerning also the pectoralis major flap that has been previously used. The margins of the defect extended from the mastoid part of the temporal bone, the tip of the lithoid bone, the hypotymbanon, revealing beneath the superior bulb of the internal jugular vein. The descending part of the facial nerve, the whole parotid gland, the maseter muscle, the exterior and interior pterygoid muscles were also invaded. The temporomandibular joint, the right condyle and coronoid process, the ramus and the body of the right mandible were included in the dissection.

The resulting defect was very large and can be characterized as hemifacial.

**Figure 2:**
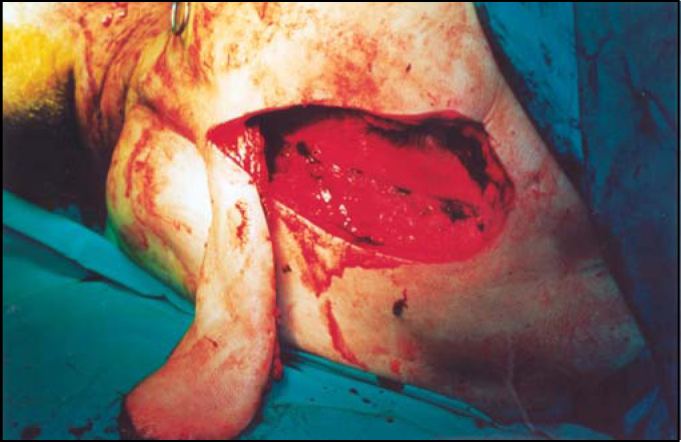
The pedicled myocutaneus lower island trapezious flap

**Figure 3:**
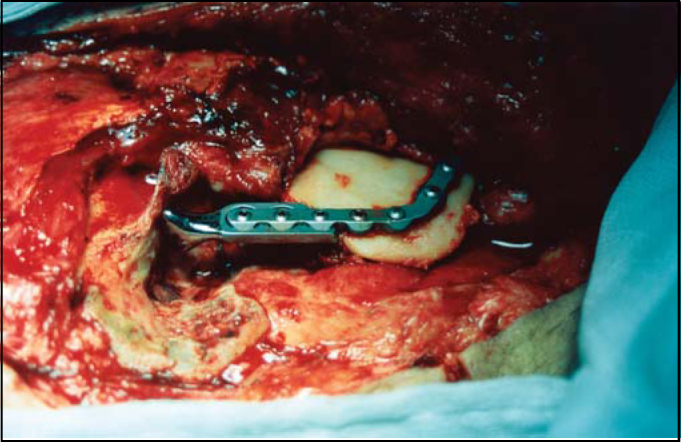
Intraoperative view. The metal condyle prosthesis for the mandible

A pedicled myocutaneous lower island trapezius flap (Figure 2) was used to cover the defect and the right temporalis muscle was transferred to cover the facial palsy. Metal right condyle prosthesis with combination of titanium reconstructive plate was used for the mandible (Figure 3). The postoperative course was uneventful and the patient returned home fifteen days after the operation.

**Figure 4:**
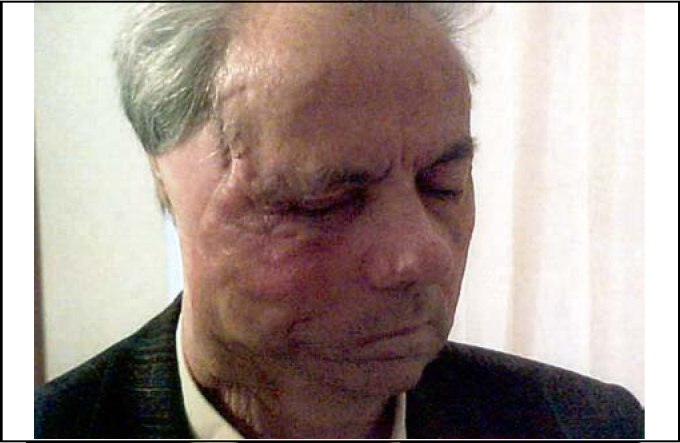
The patient, ten months postoperatively

**Figure 5:**
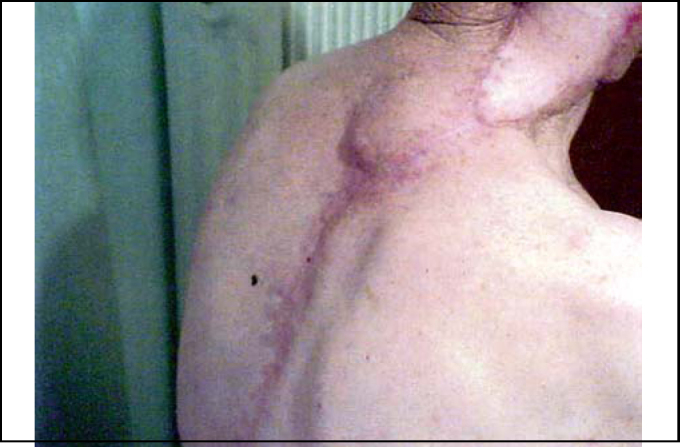
Clinical view of the donor site

**Figure 6:**
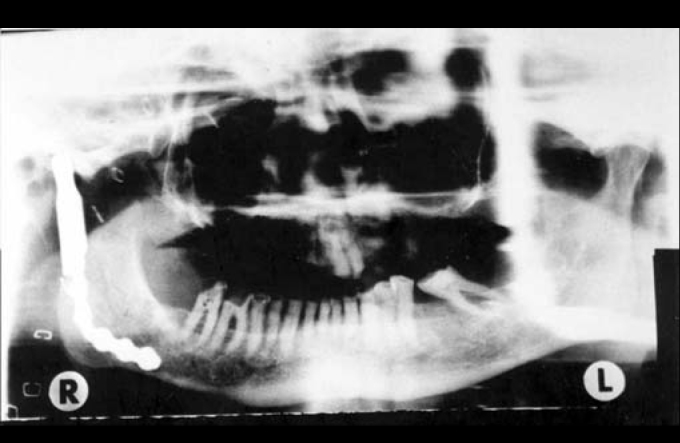
Panoramic X-ray, ten months postoperatively

**Figure 7:**
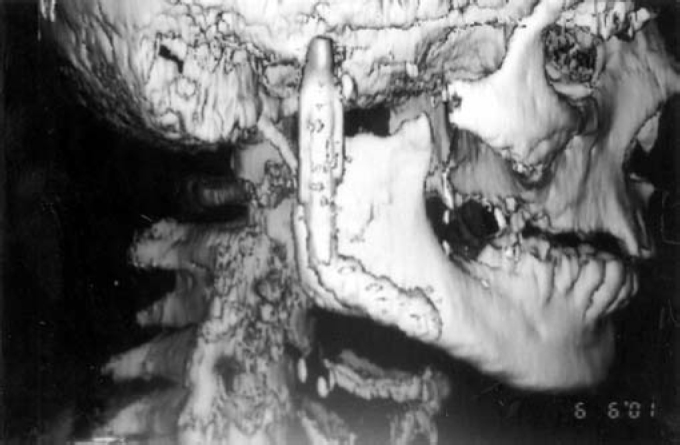
3D C/T view, ten months postoperatively

Ten months after the last operation he was fully rehabilitated (Figure 4), with normal chewing function, speaking function and acceptable esthetic results of the face and donor site (Figure 5). The panoramic X-ray (Figure 6) showed the metal prosthesis in place and the 3D C/T examination postoperatively showed the physical contour of the right mandible (Figure 7)

## DISCUSSION

Malignant tumors of the external ear and the parotid glad sometimes cause extensive damage to adjacent structures, such as temporal bone, lithoid bone, facial nerve, malar bone, maseter muscles, temporomandibular joint, mandible. Very often these tumors invase the skin and so the challenge the surgeon comes up concerning the reconstruction of these cases is huge.

Radiotherapy and neglected cases or careless surgeries make the problem even bigger.

The main concern is the better quality of life in these patients providing them functional and esthetic results.

Local and regional flaps have been used for closure of such defects but when there is quite a loss of soft tissue these flaps are of no use^9^, ^5^.

Usually a pectoralis major flap is used to close massive defects concerning large amounts of soft tissue^9^, ^5^ as the main problem is the loss of soft tissue bulk.

If the mandible is also resected, reconstructive plate or bone grafts had to be used to provide substance and profile and also mastication, deglutition, phonation^10^, ^11^.

There are several reports in the literature on the therapeutic results after the use of pectoralis major flap^4^, ^5^, ^12^ and its advantages are numerous, but when it is not indicated or cannot be used as in our case because we already used it in the first surgery and now it was invaded, lattisimus dorsi myocutaneous flap or a trapezious one are in our opinion the second best choice. We used the lower island of the trapezious flap as it offers enough skin and muscle to enable large head and neck defects^9^, ^5^ and it's easy to raise. The esthetical result is acceptable concerning the skin colors, and it can be improve by skin tattooing^5^.

This flap sometimes needs debulking, although the muscle flap atrophies over a period time.

The trapezious myocutaneous flap is very useful because it can fill up the oral cavity, nasopharynx, middle ear, an open temporomandibular joint or other contaminated cavities. The better use is with micovascular anastomosis but the requiring time is a disadvantage especially in older patients. Although pedicled flap, because of the repositioning of the patients is not preferred by some authors^5^, we thing that it can be of a great use, giving solutions in complicated cases.

When the therapeutic treatment includes a partial mandibulectomy, a bone graft especially a rib costochondral graft is suggested in the literature, but because of the need of two teams of surgeons, expanded surgery time and possible complications, we preferred the use of Martin titanium reconstructive plates and metal condyle prosthesis properly adjusted for the patient. The results from the use of titanium reconstructive plates are very encouraging in large series of patients^10^, ^11^, ^13^, ^14^. Koch and co authors in a retrospective review of 40 patients promote the titanium reconstructive plate as a permanent method of mandibular reconstruction.

These plates maintain jaw contour and occlusal relationships of residual teeth. The acceptable results rates of 73% to 93%^10^, ^13^, ^14^ and there is failure rate of 33,3%^10^.

Complications related to the use of flaps and plates in oromandibular reconstruction are usually, infection, intraoral exposure of the plate, (that needs plate removal, or local flap coverage). Also external exposure or bar fracture can be detected^10^, ^13^, ^14^.

Our patient eight months postoperatively showed an infection in the temporal region, which after antibiotic treatment with ciproxin for two months had been eliminated.

In conclusion, a number of reconstructive options are available for large defects of temporal bone and parotid area, which needs large amounts of tissue. The use of trapezious flap with combination of temporal muscle and reconstructive titanium plate for the mandible gives acceptable results and quality of life in cancer patients.
